# Genetic structure of *Plasmodium vivax *and *Plasmodium falciparum *in the Bannu district of Pakistan

**DOI:** 10.1186/1475-2875-9-112

**Published:** 2010-04-23

**Authors:** Lubna Khatoon, Frederick N Baliraine, Mariangela Bonizzoni, Salman A Malik, Guiyun Yan

**Affiliations:** 1 Department of Biochemistry, Faculty of Biological Sciences, Quaid-i-Azam University, Islamabad, Pakistan; 2 Department of Medicine, Division of Infectious Diseases, University of California - San Francisco, P.O. Box 0811, San Francisco, CA 94143-0811, USA; 3 College of Health Sciences, Program in Public Health, University of California - Irvine, Irvine CA 92697-4050, USA

## Abstract

**Background:**

*Plasmodium vivax *and *Plasmodium falciparum *are the major causative agents of malaria. While knowledge of the genetic structure of malaria parasites is useful for understanding the evolution of parasite virulence, designing anti-malarial vaccines and assessing the impact of malaria control measures, there is a paucity of information on genetic diversity of these two malaria parasites in Pakistan. This study sought to shed some light on the genetic structure of *P. vivax *and *P. falciparum *in this understudied region.

**Methods:**

The genetic diversities of *P. vivax *and *P. falciparum *populations from the densely populated, malaria-endemic Bannu district of Pakistan were evaluated by analysis of their merozoite surface protein (*msp*) genes by PCR-RFLP. Specifically, the *Pvmsp-3α *and *Pvmsp-3β *genes of *P. vivax *and the *Pfmsp-1 *and *Pfmsp-2 *genes of *P. falciparum *were analysed.

**Results:**

In *P. vivax*, genotyping of *Pvmsp-3α *and *Pvmsp-3β *genes showed a high level of diversity at these loci. Four distinct allele groups: A (1.9 kb), B (1.5 kb), C (1.2 kb), and D (0.3 kb) were detected for *Pvmsp*-*3α*, type A being the most prevalent (82%). Conversely, amplification of the *P. vivax msp*-*3β *locus produced two allele groups: A (1.7-2.2 kb, 62%) and B (1.4-1.5 kb, 33%), with 5% mixed-strain infections. Restriction analysis of *Pvmsp-3α *and *Pvmsp-3β *yielded 12 and 8 distinct alleles, respectively, with a combined mixed genotype prevalence of 20%. In *P. falciparum*, all three known genotypes of *Pfmsp-1 *and two of *Pfmsp-2 *were observed, with MAD20 occurring in 67% and 3D7/IC in 65% of the isolates, respectively. Overall, 24% *P. falciparum *samples exhibited mixed-strain infections.

**Conclusion:**

These results indicate that both *P. vivax *and *P. falciparum *populations in Pakistan are highly diverse.

## Background

Malaria is a major threat to the public health and economic development of many nations. While *Plasmodium falciparum *causes most malaria-induced mortality worldwide, *Plasmodium vivax *is the major cause of malaria morbidity outside Africa [1]. *Plasmodium vivax *has historically been largely neglected in control, partly because of its lower virulence than *P. falciparum *[2]. However, recent studies from Indonesia, Papua New Guinea, Thailand and India have shown that as high as 21-27% of patients with severe malaria have *P. vivax *monoinfection [3].

Pakistan is endemic for both *P. vivax *and *P. falciparum *malaria [4,5]. The prevailing extensive agricultural practices, an expansive irrigation network, and the monsoon rains act together to promote a favourable environment for malaria transmission in many areas of Pakistan. According to the World Health Organization (WHO), 97% (approximately 150 million) of the Pakistani population is at risk of contracting malaria, with an estimated nationwide burden of 1.6 million cases per year [6]. Malaria transmission in Pakistan is markedly seasonal and prone to epidemic outbreaks in particular geographical areas, especially the North-West Frontier Province (NWFP), the Balochistan province and the Sindh province [7]. The main malaria transmission season is September through November, following the monsoon season. However, malaria transmission occurs perennially along the Western border and coastal areas of the country. There is a brief transmission season during spring (March-April), but most of the spring cases are believed to be delayed expressions of infections acquired after the monsoon season or relapsing *P. vivax *malaria [7]. Overall, *P. vivax *accounts for 75%, while *P. falciparum *accounts for 25% of the malaria burden in Pakistan [7].

*Plasmodium vivax *infection is usually treated with chloroquine, but parasites are often inadvertently exposed to sulphadoxine-pyrimethamine, the commonly used drug for chemoprophylaxis and treating uncomplicated falciparum malaria. Indeed, high frequencies of antifolate resistance-conferring mutations are detected in the *P. vivax *dihydropteroate synthase (*Pvdhps*) and dihydrofolate reductase (*Pvdhfr*) genes [8-11]. The emergence of drug resistance and increased virulence in *P. vivax *warrants the attention of public health practitioners to both species. Parasite diversity plays a key role in parasite's survival strategies, including the potential for recombination, clonal expansion, gametocyte production, drug resistance, and immune response evasion [12,13]. Studies of the population structure of malaria parasites are, therefore, essential for understanding the evolution of parasite virulence and the role of parasite diversity in malaria transmission, and for designing control tools, including vaccines, as well as evaluating the impact of malaria control measures [14,15].

In this study, the genetic diversities of *P. falciparum *and *P. vivax *isolates from a malaria-endemic region in the North-West Frontier Province of Pakistan were analysed. Merozoite surface protein (*msp*) is one of the proteins of the erythrocytic stage of the parasite's life cycle. *Plasmodium falciparum msp-1, msp-2*, and *P. vivax msp-3 *are involved in helping the parasite escape the host's immune responses, and are potential targets for vaccine development [16]. *Pfmsp-1 *has three distinct allelic families: K1, MAD20 and RO33, while *Pfmsp-2 *has two distinct families, 3D7/IC and FC27 [17]. *Pvmsp*-*3*, particularly *Pvmsp-3α*, is a candidate molecule for inclusion in a *P. vivax *vaccine [16,18]. *Pvmsp-3α *and *Pvmsp-3β *have also been used as markers in population genetic studies worldwide [19-22]. The best strategy for detecting genetic diversity is to analyse more than one marker gene, because then the probability that different clones share the same genotype by chance is substantially reduced [23]. Accordingly, it is suggested that analysis of both *Pvmsp-3α *and *Pvmsp3β *genes of *P. vivax *enables greater capacity in identifying parasite haplotypes and detecting mixed strain infections [19,20]. For the same reason, both *Pfmsp-1 *and *Pfmsp-2 *genes are often analysed in the case of *P. falciparum*.

Since immunity to malaria is strain-specific, characterization of polymorphism in *msp *antigens among natural populations in different geographical regions should be conducted to identify the widest possible range of potential vaccine targets [14,24]. While genetic diversity studies involving vaccine-candidate genes have been widely carried out in neighbouring India [14], Iran [25], Afghanistan [26] and other parts of the world [24], there is a dearth of information on the genetic structure of *P. vivax *and *P. falciparum *in Pakistan [27]. This study sought to examine genetic polymorphisms among Pakistani malaria isolates and compare diversities and allele types with those reported from other parts of the world. Specifically, polymorphisms of the *Pvmsp-3α *and *Pvmsp-3β *genes in *P. vivax*, and *Pfmsp-1 *and *Pfmsp-2 *genes in *P. falciparum *isolates from Pakistan were assessed.

## Methods

### Study area, sample collection and DNA preparation

**Figure 1:**
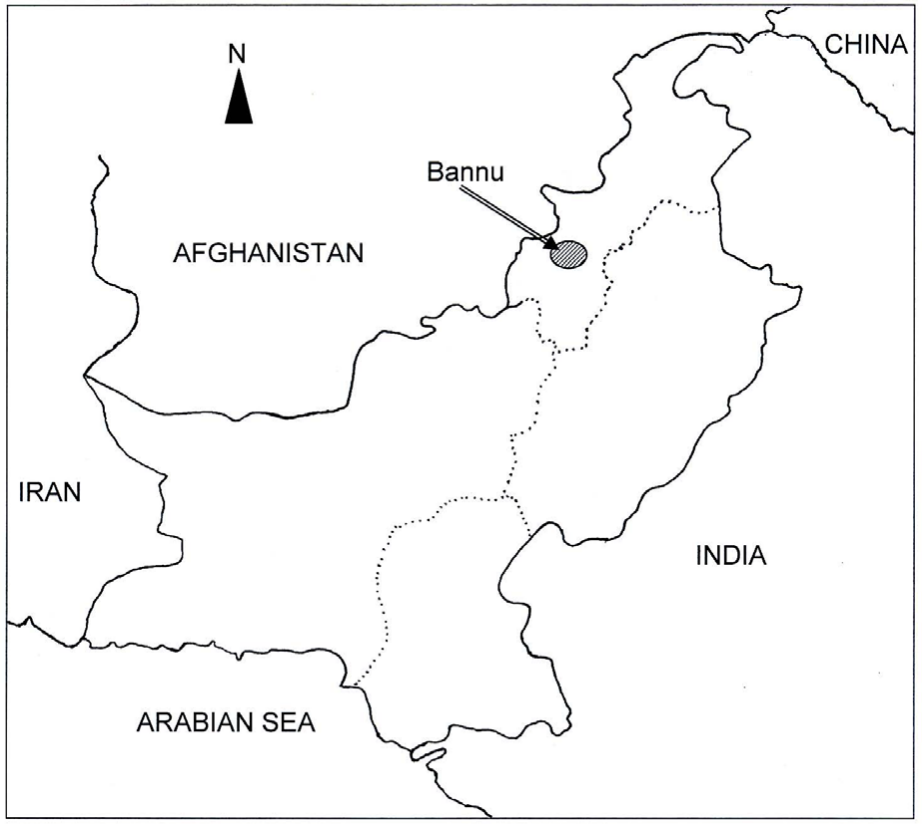
**A map of Pakistan showing the sampling area**. The map is not drawn to scale.

The study was carried out in Bannu district (32 ° 43' - 33 ° 06' N; 70 ° 22' - 57' E, Figure 1), North-West Frontier Province (NWFP), Pakistan. This populous district (552 persons/Km^2^) is listed among the most malaria-afflicted areas in Pakistan [4]. The Kurram and Gambila rivers traverse Bannu district and provide water for irrigation as well as, inevitably, forming breeding grounds for malaria vectors. The main mosquito vectors are *Anopheles culicifacies *and *Anopheles stephensi *[7,28], and the annual parasite incidence (API) is in the range of 1.6 - 3.5 per 1000 population, which is substantially above the national average (0.8 per 1,000 population) [7]. Mean daily temperatures range between 10.8°C - 32.9°C. The area experiences two rainy seasons: in March and during the summer monsoon that occurs in July and August. Malaria transmission peaks following the monsoon season [28]. The district has great economic significance, being the central market of the Southern Region, in addition to serving as a safe shortcut to markets in Central Asia.

Participants were recruited from the Bannu Women and Children Hospital (BWCH) from July through October 2007. The hospital's catchment area covers the entire district. Inclusion criterion was consenting to participate in the project by symptomatic malaria patients visiting the Malaria Control Program of BWCH, irrespective of age or sex, while exclusion criterion was non-consent. After obtaining informed consent, ~200 μl of blood was collected on Whatman 3MM filter papers by finger-prick. Concurrently, thick and thin smears were prepared and stained with Giemsa for subsequent microscopic examination. Parasite DNA was extracted from filter papers using the Chelex method and infections diagnosed by a species-specific nested polymerase chain reaction (PCR) method as previously reported [29]. Positive controls were MR4 clones MRA-340G, MRA-343G, 3D7 and HB3, while sterile water was used as the negative control. Previous work on the same samples indicated that PCR is superior to microscopy in diagnosing malaria [8]. The PCR results based on these samples showed that 1.8%, 60.5%, 22.8% and 14.9% of the samples (n = 114) had *P. falciparum *only, *P. vivax *only, mixed vivax-falciparum infections, and parasite-free, respectively, while the microscopy method misdiagnosed ~15% of the cases and also failed to detect mixed-species infections [8]. The same samples have been utilized to study the population structure of the two main malaria parasite species in the Bannu district of Pakistan. The study was approved by the Institutional Review Board of Quaid-i-Azam University, Pakistan.

### PCR-RFLP analysis of *Pvmsp-3α *and *Pvmsp-3β *genes in *P. vivax*

Amplification of *Pvmsp-3α *and *Pvmsp-3β *genes of *P. vivax *and restriction digestion of the resultant amplicons were carried out following previously reported protocols [19,20,30], with some modifications. Briefly, using primer sets and cycling conditions listed in Table 1, nested PCR was used to amplify the *Pvmsp-3α *gene, in 25.0 μl reaction volumes. The primary reaction mix comprised of 19.0 μl of 1.1× PCR master mix (Thermo Scientific ABgene, Rochester, NY); 1.0 μl of each primer (10 μM), 0.5 μl of MgCl_2 _(25 mM), 0.5 μl of Taq polymerase (5 U/μl; Invitrogen, Carlsbad, CA) and 3.0 μl of DNA template. The secondary reaction comprised of 18.0 μl of 1.1× PCR master mix, 2.0 μl of each primer (10 μM), 0.5 μl of MgCl2 (25 mM), 0.5 μl of Taq polymerase (5 U/μl) and 2.0 μl of the primary reaction amplicon.

**Table 1:** Sequences of the primers used to amplify the *Pvmsp-3α *and *Pvmsp-3β *genes of *Plasmodium vivax *and the *Pfmsp-1 *and *Pfmsp-2 *genes of *Plasmodium falciparum *isolates from Bannu district, Pakistan.

**Gene (PCR reaction)**^**§**^	Primers*	PCR cycling conditions**	Reference
*P. vivax*			

*Pvmsp-3α *(N1)	F: 5'-CAGCAGACACCATTTAAGG-3'	95°C 3 min/[94°C 30 s, 54°C 30 s, 68°C 2.5 min] × 35 cycles, 68°C 5 min	[19]
	R: 5'-CCGTTTGTTGATTAGTTGC-3'		
*Pvmsp-3α *(N2)	F: 5'-GACCAGTGTGATACCATTAACC-3'	95°C 3 min/[94°C 30 s, 55°C 30 s, 68°C 2.5 min] × 30 cycles, 68°C 5 min	[19]
	R: 5'-ATACTGGTTCTTCGTCTTCAGG-3'		
*Pvmsp-3β *(N1)	F: 5'-GTATTCTTCGCAACACTC-3'	95°C 3 min/[94°C 30 s, 54°C 30 s, 68°C 2.5 min] × 35 cycles, 68°C 5 min	[20]
	R: 5'-CTTCTGATGTTATTTCCAG-3'		
*Pvmsp-3β *(N2)	F: 5'-CGAGGGGCGAAATTGTAAACC-3'	95°C 3 min/[94°C 30 s, 55°C 30 s, 68°C 2.5 min] × 30 cycles, 68°C 5 min	[20]
	R: 5'-GCTGCTTCTTTTGCAAAGG-3'		

*P. falciparum*			

*Pfmsp-1 *(N1)	F: 5'-CTAGAAGCTTTAGAAGATGCAGTATTG-3'	95°C 5 min/[94°C 30 s, 55°C 30 s, 72°C 1 min] × 35 cycles, 72°C 5 min	[17]
	R: 5'-CTTAAATAGTATTCTAATTCAAGTGGATCA-3'		
K1 family (N2)	F: 5'-AAATGAAGAAGAAATTACTACAAAAGGTGC-3'	95°C 5 min/[94°C 30 s, 56°C 30 s, 72°C 45 s] × 30 cycles, 72°C 5 min	[17]
	R: 5'-GCTTGCATCAGCTGGAGGGCTTGCACCAGA-3'		
MAD20 family (N2)	F: 5 '-AAATGAAGGAACAAGTGGAACAGCTGTTAC-3 '	95°C 5 min/[94°C 30 s, 56°C 30 s, 72°C 45 s] × 30 cycles, 72°C 5 min	[17]
	R: 5 '-ATCTGAAGGATTTGTACGTCTTGAATTACC-3 '		
RO33 family (N2)	F: 5 '-TAAAGGATGGAGCAAATACTCAAGTTGTTG-3 '	95°C 5 min/[94°C 30 s, 56°C 30 s, 72°C 45 s] × 30 cycles, 72°C 5 min	[17]
	R: 5 '-CATCTGAAGGATTTGCAGCACCTGGAGATC-3 '		
*Pfmsp-2 *(N1)	F: 5 '-ATGAAGGTAATTAAAACATTGTCTATTATA-3 '	95°C 5 min/[94°C 30 s, 56°C 30 s, 72°C 45 s] × 30 cycles, 72°C 5 min	[17]
	R: 5'-CTTTGTTACCATCGGTACATTCTT-3'		
FC27 family (N2)	F: 5'-AATACTAAGAGTGTAGGTGCARATGCTCCA-3'	95°C 5 min/[94°C 30 s, 56°C 30 s, 72°C 45 s] × 30 cycles, 72°C 5 min	[17]
	R: 5 '-TTTTATTTGGTGCATTGCCAGAACTTGAAC-3 '		
3D7/IC family (N2)	F: 5 '-AGAAGTATGGCAGAAAGTAAKCCTYCTACT-3 '	95°C 5 min/[94°C 30 s, 56°C 30 s, 72°C 45 s] × 30 cycles, 72°C 5 min	[17]
	R: 5 '-GATTGTAATTCGGGGGATTCAGTTTGTTCG-3 '		

^§ ^N1 = Nest 1 (Primary) reaction; N2 = Nest 2 (Secondary) PCR reaction

* F= Forward primer; R = Reverse primer. The reference sources of the primers are indicated.

**The cycling conditions have been modified in the present work.

For RFLP analysis, 6 μl of each PCR product was digested with 2 units of *Hha*I and *Alu*I for *Pvmsp-3α*, and *Pst *I for *Pvmsp-3β*, in 16 μl reaction volumes at 37°C for 4 hrs, following the enzyme manufacturer's protocol (New England Biolabs, Ipswich, MA). Two enzymes (*Hha*I and *Alu*I) were used for *Pvmsp-3α *because this strategy has been shown to improve the sensitivity of detecting diversity at this locus [19,31]. Specifically, as clearly illustrated by the works of Bruce *et al *[19] and Kim *et al *[31], owing to differences in discriminatory power, it is possible for the restriction pattern of one enzyme to show two isolates as being identical, while the RFLP patterns of the second enzyme reveals two different alleles; in which case the combined tally of the number of alleles would be two, rather than one.

Except for the primers (Table 1), the same primary and secondary conditions as those for *Pvmsp-3α *were used for the amplification of *Pvmsp-3β*. DNA fragments were visualized under UV illumination after electrophoresis in ethidium bromide-stained 1.8% agarose gels. Alleles were classified based on undigested PCR product size and RFLP banding patterns [20,21,32]. For *Pvmsp-3α *the major expected size polymorphisms are: type A (~1.8 - 1.9 kb), type B (~1.4 - 1.5 kb) and type C (~1.1 - 1.2 kb) [27,31-33]. Conversely, apart from type C which is substantially smaller (~0.65 kb), the expected undigested type A (1.7 - 2.2 kb) and type B (1.4 - 1.5 kb) alleles for *Pvmsp-3β *[20] are in similar size ranges to those of *Pvmsp-3α*. Mixed-clone infections were diagnosed when amplification of a single sample resulted in PCR products of different sizes, or where the summation of the lengths of the digested amplicons exceeded the size of the undigested PCR product [21]. To score the combined frequency of mixed-clone infections based on restriction data for both *Pvmsp-3α *and *Pvmsp-3β *genes, a sample which showed mixed genotypes at both genes was scored as mixed, while a sample which was monoclonal at one gene but mixed at the other was also scored as mixed in the final tally. Only if a sample was monoclonal at both genes was it counted as monoclonal in the final, combined data tally.

### PCR analysis of *Pfmsp-1 *and *Pfmsp-2 *genes in *P. falciparum*

*Plasmodium falciparum *genotyping was carried out on the polymorphic block 2 of the *msp-1 *gene on chromosome 9, and on block 3 of the *msp-2 *gene on chromosome 2, using previously reported nested PCR conditions [17], with some modifications. In the primary reaction, primer pairs corresponding to the conserved sequences spanning the polymorphic regions of the two genes were included (Table 1). For a total reaction volume of 25 μl, 20.2 μl of 1.1× PCR master mix (Thermo Scientific ABgene, Rochester, NY) was used, together with 0.25 μl of 10 μM of each of primer, 0.3 μl of Taq polymerase (5 U/μl), 1.0 μl of 25 mM MgCl_2 _and 3.0 μl of DNA extract. Using the product generated in the first reaction as a template, five separate secondary reactions were performed using, in each case, a family-specific primer pair (Table 1) in order to determine the presence of allelic variants from the MAD20, Kl and RO33 families of *msp-1*, and the FC27 and 3D7/IC families of *msp-2*. In the secondary reaction, 21.2 μl of 1.1× PCR master mix were used along with 0.25 μl of 10 μM of each of primer, 1.0 μl of 25 mM MgCl_2_, 0.3 μl of Taq polymerase (5 U/μl) and 2.0 μl of the primary reaction PCR product. Products were run on ethidium bromide-stained 2.0% agarose gels, and visualized under UV light. The allelic variants of the *P. falciparum *isolates were easily resolved on the agarose gels, because the primers used are strictly specific to the respective *Pfmsp *families, and they bear the additional advantage of not amplifying with other Plasmodium species or human DNA [17]. In all cases, gel documentation was carried out using Kodak Digital Science 1D Image Analysis Software version 2.03.

## Results

A total of 114 blood samples were collected during the four-months sampling period, but 17 samples were excluded from further analysis after being confirmed to be parasite-free by PCR analysis. The participants (42 female, 72 male), aged 1-60 years, were all local, Pakistani residents of Bannu, with no known history of travel outside Bannu or anti-malarial treatment within two weeks prior to blood sampling.

### Size polymorphism of *P. vivax Pvmsp-3α *and *Pvmsp-3β *genes

**Figure 2:**
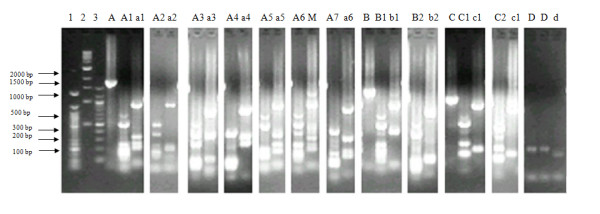
**Major merozoite surface protein-3α alleles identified by PCR and digestion with *Alu *I and *Hha *I restriction enzymes in the *Plasmodium vivax *population from Bannu district, Pakistan**. Lane 1 = 50 bp, 2 = 1 Kb, and 3 = 100 bp DNA marker. A, B, C = Undigested PCR products. A1, A2 ... are allele types revealed by digestion of the respective PCR products with *Alu*I, while a1, a2 ... are the allele types obtained by digestion with *Hha*I, and M = mixed genotype. Frequencies of these alleles are presented in Table 2.

Fifty *P. vivax *samples successfully amplified at *Pvmsp-3α*. Four distinct allele size polymorphisms were observed at *Pvmsp-3α*, with 82% (41/50) isolates having allele sizes 1.9 kb (type A), 6% (3/50) with the 1.5 kb fragment (type B), 8% (4/50) with the 1.2 kb fragment (type C), 2% (1/50) with the 0.3 kb fragment (type D) (Figure 2). One isolate (2%) exhibited mixed-strain infection. To obtain a higher resolution on infection diversity, the PCR fragments were digested the using *Alu*I and *Hha*I enzymes. Restriction digestion of the *Pvmsp-3α *PCR product with *Alu*I yielded 12 different alleles designated as A1-A7, B1, B2, C1, C2 and D (Figure 2); allele A3 being the most abundant (24%; Table 2). Conversely, restriction with *Hha*I produced 10 alleles denoted as A1-A6, B1, B2, C1, and D (Figure 2); allele A5 being the most abundant (22%). Two samples (4%) remained undigested by *Hha*I, while 6% isolates showed mixed-strain infections (Table 2). A comparative analysis of both *Alu*I and *Hha*I RFLP patterns showed a total of 12 different alleles in the 50 samples typed at the *Pvmsp-3α *locus, with mixed-strain infections occurring in 8% (4/50) of the samples.

**Table 2:** Distribution of *Plasmodium vivax msp-3α *allele frequencies as determined by the PCR-RFLP method.

	Digestion with *Alu*I (n = 50)		Digestion with *Hha*I (n = 50)			Digestion with *Pst*I (n = 39)	
				
Gene	Allele	Frequency	Allele	Frequency	Gene	Allele	Frequency
msp-3α*	A1	8% (4/50)	A1	8% (4/50)	*msp-3β*	A1	8% (3/39)
	A2	20% (10/50)	A2	12% (6/50)		A2	13% (5/39)
	A3	24% (12/50)	A3	20% (10/50)		A3	13% (5/39)
	A4	8% (4/50)	A4	6% (3/50)		A4	10% (4/39)
	A5	12% (6/50)	A5	22% (11/50)		A5	5% (2/39)
	A6	6% (3/50)	A6	6% (3/50)		B1	8% (3/39)
	A7	4% (2/50)	B1	4% (2/50)		B2	5% (2/39)
	B1	2% (1/50)	B2	4% (2/50)		B3	5% (2/39)
	B2	4% (2/50)	C1	6% (3/50)		U	15% (6/39)
	C1	2% (1/50)	D	2% (1/50)		M	18% (7/39)
	C2	6% (3/50)	U	4% (2/50)			
	D	2% (1/50)	M	6% (3/50)			
	M	2% (1/50)					

*Digested with two enzymes in order to improve the sensitivity of detecting diversity at this locus [19].

U: Uncut

M: Mixed genotype

**Figure 3:**
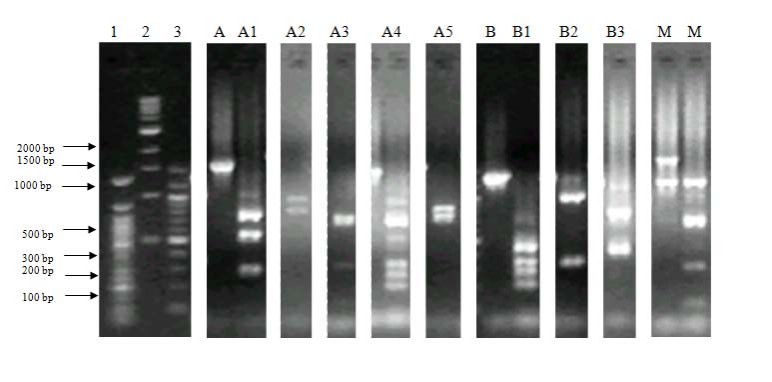
**Major merozoite surface protein-3β alleles identified by PCR and digestion with *Pst *I restriction enzymes in the *Plasmodium vivax *population from Bannu district, Pakistan**. Lane 1 = 50 bp, 2 = 1 Kb, and 3 = 100 bp DNA marker. A, B, C = Undigested PCR products. A1, A2 ... are allele types revealed by digestion of the respective PCR products with *Pst*I, while M = mixed genotype. Frequencies of these alleles are presented in Table 2.

Thirty-nine *P. vivax *samples amplified for the *Pvmsp-3β *gene. Amplification resulted in two major allele polymorphisms, type A (1.7-2.2 kb) in 62% isolates, and type B (1.4-1.5 kb) in 33% isolates, while 5% of the isolates had mixed genotypes. A total of 8 restriction patterns, designated A1-A5 and B1-B3, were obtained following amplicon digestion with *Pst*I (Figure 3 and Table 2). Alleles A2 (13%; 5/39) and A3 (13%; 5/39) were the most prevalent among the samples analysed at *Pvmsp-3β*. Restriction analysis of the PCR products provided a higher resolution of the infection complexity, revealing that 18% of the samples analysed at the *Pvmsp-3β *gene exhibited mixed-strain infection, up from the 5% mixed infections detected without restriction analysis (Table 2). Overall, 10 out of the 50 *P. vivax *samples analysed (20%) had mixed-strain infections (three detected by *Pvmsp-3α *analysis only, six by *Pvmsp-3β *analysis only, and one detected as mixed in both genes).

### Size polymorphism of *P. falciparum Pfmsp-1 *and *Pfmsp-2 *genes

A high level of diversity was observed among *P. falciparum *samples. Overall, 9 *Pfmsp-1 *alleles and 10 *Pfmsp-2 *alleles were detected. At the *Pfmsp-1 *locus, 2 K1 family alleles (170-220 bp), 6 MAD20 alleles (100-250 bp), and 1 RO33 allele (200 bp) were obtained. One of the *P. falciparum *samples did not amplify at either locus despite repeated amplification, hence 25 samples were genotyped. Of these, 84% (21/25) samples successfully amplified at *Pfmsp-1*, while 92% (23/25) samples amplified at *Pfmsp-2*. A total of 19% (4/21) samples exhibited mixed-strain infections at *Pfmsp-1 *(Table 3).

**Table 3:** Allelic distribution of *Pfmsp-1 *and *Pfmsp-2 *among Pakistani *Plasmodium falciparum *isolates from Bannu district, Pakistan.

Gene*	Genotype	Frequency
*Pfmsp-1 *(n = 21)	K1	5% (1/21)
	MAD20	67% (14/21)
	RO33	9% (2/21)
	K1+MAD20	14% (3/21)
	MAD20+RO33	5% (1/21)
*Pfmsp-2 *(n = 23)	3D7/IC	65% (15/23)
	FC27	26% (6/23)
	FC27+3D7/IC	9% (2/23)

*A total of 25 samples were genotyped. All except two of the samples that amplified at *Pfmsp-1 *were amplified at the *Pfmsp-2 *gene.

At locus *Pfmsp-2*, three FC27 alleles (250-500 bp) and seven 3D7/IC alleles (400-800 bp) were detected. The 3D7/IC genotype was predominant, constituting 65% (15/23) of the samples, while 9% (2/23) of the samples exhibited mixed-strain infections at the *Pfmsp-2 *gene (Table 3). All except two of the samples that amplified at *Pfmsp-1 *were successfully amplified at *Pfmsp-2*. A comparative analysis of both the genotype data for *Pfmsp-1 *and *Pfmsp-2 *genes showed that overall, 24% (6/25) *P. falciparum *samples exhibited mixed-strain infections.

## Discussion

Malaria is a major health problem in Pakistan, and the situation is not helped by the fact that it shares borders with countries like Afghanistan, India and southern Iran, where the disease is also highly endemic. Moreover, there is substantial cross-border human migration within this region, exacerbated by upheavals in places like Afghanistan that lead to refuge influxes, all of which facilitate malaria transmission. The introduction of new alleles by mutation, migration and recombination generates genetic diversity in natural populations, while the immune responses of the human host, as well as chemotherapy play key roles in selection, which affects the frequency of new alleles in parasite populations [33]. Analyses of genetic diversity can therefore give important clues on a parasite's response to human interventions like drugs or vaccines, because directional selection will favour the fixation of advantageous alleles in the population, thereby reducing genetic diversity [34]. Despite its importance, data on the genetic diversity of the two key malaria parasite species circulating in Pakistan are largely lacking, and there is only one recent study analysing the diversity of *P. vivax *in the Federally Administered Tribal Areas (FATA) of the country [27]. The need for detailed studies of the nature and extent of Plasmodium population diversity in this region is, therefore, obvious.

The study participants were all local, Pakistani residents of Bannu, with no known history of travel outside the district, but fact that the area has a high influx of refugees from Afghanistan as well as being a commercial hub for central Asia cannot preclude them from being infected with diverse parasite strains from other geographical areas. Moreover, while it cannot be stated with absolute certainty that none of the participants had exposure to anti-malarial drugs, because no drug assays were carried out, there was no sufficient reason to expect self medication, since the patients had access to free diagnosis and treatment through the Malaria Control Program at the Bannu Hospital. Moreover, relatively high levels of infection complexity were observed, implying that any possible contribution of drugs to reducing the observed diversity may have been insignificant. In particular, high levels of polymorphism were observed in the potential vaccine candidate antigens in *P. vivax *and *P. falciparum *isolates from Bannu. In this study, four distinct sizes of PCR products (A, B, C and D) for *Pvmsp-3α *were detected, while in Thailand [21], Afghanistan [26], and the FATA of Pakistan [27], only 3 allele sizes (A, B and C) were obtained for this gene. The present finding, by *Alu*I digestion of *Pvmsp-3α *amplicons, that allele group A was most prevalent is consistent with observations from the FATA of Pakistan [27], Venezuelan Amazon [33], Papua New Guinea [19], Afghanistan and southern Iran (with which Pakistan shares a common border); but it differs from hypo-endemic northern Iran where type C of the *Pvmsp-3α *gene is predominant [26,32]. Overall, like in Iran and Afghanistan, the *Pvmsp-3α *gene was highly polymorphic in Pakistan. In addition to the three major alleles, smaller bands (0.45 - 0.57 kb) have occasionally been amplified in *P. vivax *strains from other parts of Asia [18]. A smaller (0.3 kb) allele was detected in Pakistan.

The PCR-RFLP patterns for *P. vivax Pvmsp-3β *gene produced 2 categories (A [1.7-2.2 kb] and B [1.4-1.5 kb]) of size polymorphisms, in contrast to Northwest Thailand where three distinct allele types (A, B and C [~0.65 kb]) were found [20]. Moreover, 15% of the Pakistani isolates belonging to allele type B did not have any restriction site for *Pst*I, in contrast to Thailand and China where all type B isolates were digested by *Pst*I [20]. Data from PCR-RFLP of the *Pvmsp-3α *and *Pvmsp-3β *locishowed that 20% of the Pakistani *P. vivax *isolates exhibited mixed-strain infections, which is three-fold higher than what was observed in Bengbu (China), and is comparable to sites in Thailand (21%) [20], but lower than the complexity (30%) observed in the FATA region of Pakistan which directly shares a border with Afghanistan [27]. In northern Iran [32] and Hongshuihe (China), no mixed genotypes were detected [20]. Apart from differences in transmission intensities in various areas, the observed differences in parasite types could be attributed to factors such as sampling biases, host immune selective pressure on particular types and/or spatiotemporal changes in the availability of different mosquito species that can transmit specific parasite types in a particular area over different times or seasons, with areas that share the same mosquito types tending to have similar parasite types [27]. As such, comparative studies of *P. vivax *parasite types as well as the mosquito species in different areas during the same season would be interesting.

This study shows high levels of polymorphism at *msp-1 *(9 alleles) and *msp-2 *(10 alleles) loci for the *P. falciparum *isolates from the Bannu district of Pakistan. Such high levels of genetic diversity at these two genes mirror what has been observed in neighbouring Iran [35] and India [36] where all three families of *msp-1 *(K1, MAD20 and RO33) and the two of *msp-2 *families (FC27 and 3D7/IC) were amplified, and the MAD20 allelic family was predominant [37-39]. However, the prevalence of the K1+MAD20 mixed genotype in India was 54.3% [39], which is substantially higher (about four-fold) than what was observed in Pakistan (14%). Furthermore, the Indian samples showed an over-representation of the 200 bp allele of MAD20 [36] whereas a shorter (150 bp) MAD20 allele was predominant in the Pakistani samples. For *msp*-2, the 3D7/IC alleles were predominant in Pakistan, just as has been reported in neighbouring Iran [35]. However, the allele sizes in the Pakistani isolates (400-800 bp for 3D7/IC alleles and 250-500 bp for FC27 alleles) were larger than those observed in Iran (400-600 bp for 3D7/IC alleles and 200-400 bp for FC27 alleles). In remarkable contrast, all *P. falciparum *isolates from the hypoendemic French Guiana (South America) were monomorphic at *msp-1 *[40] while in Colombia, *msp-1 *was monomorphic and *msp-2 *had only 3 alleles [41]. These results suggest high spatial heterogeneity of *P. falciparum *infections, which may result from geographical isolation, differences in transmission intensity, and malaria treatment policies. In particular, geographically isolated parasite populations are expected to be less diverse than exposed populations, due to lack of a continuous introduction of new parasite clones from other areas. Similarly, because of high levels of inbreeding, areas of low transmission intensity tend to have less diverse parasites than areas of high endemicity, where the rates of cross-fertilization are higher [20,42]. In the same vein, treatment policies that focus on using highly potent drugs like artemisinin combination therapies (ACTs) that kill the asexual blood stage parasites as well as gametocytes are more likely to decrease parasite transmission and clonal diversity than regimens based on drugs that act only on the blood stage parasites [43,44]. In Pakistan, monotherapies that work on blood stage parasite are still largely used, with the key first line malaria drugs being chloroquine for *P. vivax *and sulphadoxine-pyrimethamine (SP) for *P. falciparum *[4]. Moreover, recent molecular work shows that parasites in this region are rapidly developing resistance to these drugs [8], and worse still, antimalarial drugs like SP are known to trigger a massive release of gametocytes; the infective stage for the mosquito vector [44].

## Conclusions

In conclusion, this study indicates that both *P. vivax *and *P. falciparum *populations in the Bannu district of Pakistan are highly diverse. The high prevalence of mixed-species and mixed-clone infections in *P. falciparum *and *P. vivax *in Bannu likely results from high transmission and parasite introduction by human travel. Bannu is basin-like, and is intersected by two major rivers whose banks and associated irrigation activities provide ideal breeding grounds for malaria vectors. Secondly, besides being a regional commercial hub for Central Asia, the area has a high influx of Afghan refugees who may also facilitate the introduction of parasite clones from Afghanistan. Furthermore, mixed-clone infections within the human host facilitate cross-fertilization, meiotic recombination and production of novel parasite strains during the sexual stage in the mosquito vector [20,42,45]. Such heterogeneity might thus cause differences in parasite virulence, transmissibility and responses to chemotherapy, with important implications for malaria control measures in this populous region. Imperatively, further studies aimed at determining the associations between different parasite genotypes and clinical manifestations of the disease might be valuable for future management of the disease in this area of mixed-species endemicity [27].

## Competing interests

The authors declare that they have no competing interests.

## Authors' contributions

LK: Participated in the study design, carried out the molecular genetic work, data analysis and drafted the manuscript. FNB: Participated in the molecular genetic work and in the critical review of the manuscript. MB: Participated in writing of the manuscript. SAM: Participated in the study design and co-ordination of field sample collection. GY: Participated in the study design and manuscript preparation. All authors read and approved the final manuscript.
